# Serum Homocysteine Levels Correlate With Carotid Intima-Media Thickness in Adults With Subclinical Atherosclerosis: A Cross-Sectional Study

**DOI:** 10.7759/cureus.108150

**Published:** 2026-05-02

**Authors:** Sri Lakshmi Kothakapa, Parimi Vamsi Krishna, Sainithya Chittireddy, Sanjay Reddy Thanugundla, Sulekha Ramireddy, Rama Chakra Kushal Thota, Muralidhar Chinnapaka

**Affiliations:** 1 Internal Medicine, Malla Reddy Institute of Medical Sciences, Hyderabad, IND; 2 Internal Medicine, J.J.M. Medical College, Davanagere, IND; 3 General Medicine, Malla Reddy Institute of Medical Sciences, Hyderabad, IND; 4 General Medicine, Gandhi Medical College, Secunderabad, IND; 5 Pharmacology, Government Medical College, Maheshwaram, Hyderabad, IND

**Keywords:** atherosclerosis, biomarker, cardiovascular risk, carotid intima-media thickness, endothelial dysfunction, homocysteine

## Abstract

Background: Early atherosclerosis may remain clinically silent until significant vascular disease develops. Carotid intima-media thickness is a useful non-invasive measure for detecting subclinical vascular wall changes. Serum homocysteine has been linked to endothelial dysfunction, oxidative stress, and vascular inflammation. The present study was conducted with the following objectives: (1) to compare serum homocysteine levels between adults with subclinical atherosclerosis and controls; (2) to assess the correlation between serum homocysteine levels and carotid intima-media thickness; and (3) to evaluate the diagnostic performance of serum homocysteine in identifying subclinical atherosclerosis.

Methods: A cross-sectional analytical study was conducted among 120 adults aged 30-60 years. Participants were divided into two groups: individuals with subclinical atherosclerosis (n=60), identified by increased carotid intima-media thickness (CIMT >0.8 mm), and age- and sex-matched controls (n=60). Fasting serum homocysteine levels were measured using an immunoassay. CIMT was assessed by high-resolution ultrasonography. Statistical analysis included independent t-test, Pearson correlation, and receiver operating characteristic (ROC) analysis.

Results: Mean serum homocysteine levels were significantly higher in the subclinical atherosclerosis group compared to controls (18.6 ± 5.2 µmol/L vs 10.9 ± 3.7 µmol/L, p<0.001). CIMT values were also elevated in cases (0.92 ± 0.08 mm) compared to controls (0.64 ± 0.07 mm, p<0.001). A moderate positive correlation was observed between homocysteine levels and CIMT (r=0.62, p<0.001). ROC analysis showed an area under the curve (AUC) of 0.84 for homocysteine, with a cut-off value of 14.5 µmol/L yielding 78% sensitivity and 82% specificity for detecting early atherosclerosis.

Conclusion: Elevated serum homocysteine is strongly associated with early vascular changes and correlates with CIMT. It may serve as a useful, accessible biomarker for early detection of atherosclerosis in clinical settings.

## Introduction

Atherosclerosis is a chronic, progressive disorder of the arterial wall characterised by lipid deposition, inflammation, and endothelial dysfunction. It begins silently many years before the appearance of clinical manifestations such as myocardial infarction or stroke. Early identification of individuals at risk is therefore essential for timely intervention. Conventional risk factors such as hypertension, diabetes mellitus, dyslipidaemia, and smoking explain a substantial proportion of cardiovascular events, yet a considerable number of cases occur in individuals without overt risk factors. This gap has driven interest in identifying additional biochemical markers that reflect early vascular injury [1,2].

Endothelial dysfunction is considered a key initiating event in atherogenesis. It leads to impaired vasodilation, increased oxidative stress, and a pro-inflammatory state that favours plaque formation. Among emerging biomarkers, homocysteine has attracted attention due to its potential role in vascular damage. Homocysteine is a sulphur-containing amino acid formed during methionine metabolism. Under normal physiological conditions, it is either remethylated to methionine or trans-sulfurated to cysteine. Disruption of these pathways, due to genetic, nutritional, or metabolic factors, results in elevated plasma homocysteine levels, a condition known as hyperhomocysteinaemia [3,4].

Elevated homocysteine has been implicated in several mechanisms that promote atherosclerosis. It induces oxidative stress by generating reactive oxygen species, reduces nitric oxide bioavailability, and promotes endothelial cell injury. In addition, homocysteine enhances smooth muscle cell proliferation, increases platelet aggregation, and alters coagulation pathways, thereby contributing to both plaque formation and thrombosis [5-7]. These pathophysiological effects suggest that homocysteine may serve not only as a marker but also as a mediator of vascular disease.

Epidemiological studies have demonstrated a positive association between elevated homocysteine levels and cardiovascular diseases, including coronary artery disease, stroke, and peripheral vascular disease [8,9]. However, the role of homocysteine in early or subclinical atherosclerosis remains an area of ongoing investigation. Carotid intima-media thickness (CIMT), measured using high-resolution ultrasonography, is widely accepted as a non-invasive surrogate marker of early atherosclerotic changes. Increased CIMT has been shown to correlate with future cardiovascular events and is useful in identifying individuals at high risk even before clinical disease develops [10-12].

Several studies have reported a correlation between homocysteine levels and CIMT, suggesting that elevated homocysteine may reflect early structural changes in the arterial wall [13,14]. However, results have not been entirely consistent across populations, possibly due to differences in genetic background, dietary habits, vitamin status, and coexisting risk factors. In particular, deficiencies of folate, vitamin B12, and vitamin B6, which are essential for homocysteine metabolism, are common in many populations and may influence homocysteine levels and their vascular effects [15,16].

In the Indian context, cardiovascular disease is increasing at an alarming rate, often presenting at a younger age compared to Western populations. Identifying cost-effective and easily measurable biomarkers is therefore of significant clinical relevance. Serum homocysteine estimation is relatively simple and may provide additional information beyond traditional risk assessment tools. However, there is limited data evaluating its role as an early biomarker of atherosclerosis in the local population [17-19].

Given this background, the present study was planned to evaluate the relationship between serum homocysteine levels and subclinical atherosclerosis assessed by carotid intima-media thickness. The primary objective was to compare serum homocysteine levels between adults with subclinical atherosclerosis, identified by increased carotid intima-media thickness, and controls. The secondary objectives were to assess the correlation between serum homocysteine levels and carotid intima-media thickness and to evaluate the diagnostic performance of serum homocysteine in identifying subclinical atherosclerotic changes using receiver operating characteristic curve analysis. These objectives were framed to examine the possible role of serum homocysteine as a supportive marker for early vascular assessment, while avoiding any assumption of causality due to the cross-sectional nature of the study.

## Materials and methods

Study design and setting

This hospital-based cross-sectional analytical study was conducted at Malla Reddy Institute of Medical Sciences, Hyderabad, from January 2024 to December 2025. The study was designed to evaluate the relationship between serum homocysteine levels and early atherosclerotic vascular changes assessed by carotid intima-media thickness (CIMT). Since both serum homocysteine and CIMT were measured at a single point in time, a cross-sectional design was considered suitable for identifying an association between these two parameters. However, the design was not intended to establish causality or predict future cardiovascular events.

Study population

Adults aged 30 to 60 years attending the outpatient department for routine health evaluation or minor medical complaints were screened for eligibility. After clinical evaluation and carotid ultrasonography, eligible participants were classified into two groups. The study group included adults with subclinical atherosclerosis, defined by CIMT greater than 0.8 mm. The control group included apparently healthy adults with CIMT of 0.8 mm or less and no clinical evidence of cardiovascular disease. A total of 120 participants were included, with 60 participants in each group, allowing balanced comparison between individuals with and without early vascular changes.

Inclusion criteria

Participants between 30 and 60 years of age who were willing to participate and provided written informed consent were included in the study. Individuals were included in the subclinical atherosclerosis group if their CIMT was greater than 0.8 mm on carotid ultrasound examination. Participants with CIMT of 0.8 mm or less, without a history or clinical evidence of cardiovascular disease, were enrolled as controls. This grouping was done to ensure clear separation between participants with early vascular wall thickening and those without detectable subclinical atherosclerotic change.

Exclusion criteria

Participants were excluded if they had medical conditions or treatment histories that could independently alter serum homocysteine levels or affect vascular wall thickness. These included known coronary artery disease, previous myocardial infarction, stroke, transient ischaemic attack, peripheral arterial disease, chronic kidney disease, chronic liver disease, thyroid dysfunction, active infection, chronic inflammatory disorders, malignancy, and pregnancy. Individuals receiving vitamin B12, folic acid, multivitamin supplementation, lipid-lowering therapy, or drugs known to interfere with homocysteine metabolism were also excluded. These criteria were applied to reduce confounding and to improve the internal validity of the study findings.

Sample size calculation

The sample size was calculated based on the expected difference in serum homocysteine levels between adults with subclinical atherosclerosis and controls, as reported in previously published studies. The calculation was performed using a two-group comparison of means, with a statistical power of 80% and a level of significance of 5% (alpha = 0.05). The assumed effect size was derived from earlier published data showing higher mean serum homocysteine levels among individuals with increased CIMT compared with those without vascular wall thickening. Based on these assumptions, the minimum required sample size was estimated to be approximately 54 participants per group. To improve precision and to allow for incomplete data or technical exclusions, the sample size was increased to 60 participants per group. Thus, the final study sample consisted of 120 participants, with 60 in the subclinical atherosclerosis group and 60 in the control group.

Data collection procedure

After obtaining written informed consent, each participant underwent a structured clinical assessment. Demographic details, personal history, dietary pattern, smoking status, alcohol intake, physical activity, and relevant medical history were recorded using a predesigned case record form. Particular attention was given to conventional cardiovascular risk factors, including hypertension, diabetes mellitus, dyslipidaemia, family history of premature cardiovascular disease, and obesity.

Anthropometric measurements were obtained using standard procedures. Body weight was recorded using a calibrated weighing scale, and height was measured using a stadiometer. Body mass index was calculated as weight in kilograms divided by height in metres squared. Blood pressure was measured in the seated position after at least five minutes of rest, using an appropriately sized cuff. Two readings were taken at an interval of five minutes, and the average value was used for analysis. These baseline variables were recorded to describe the study population and to assess the distribution of cardiovascular risk factors between the two groups.

Biochemical analysis

Fasting venous blood samples were collected from all participants after an overnight fast of eight to 10 hours. Under aseptic precautions, approximately 5 mL of venous blood was drawn from the antecubital vein into a plain vacutainer tube. The samples were allowed to clot at room temperature and were then centrifuged at 3,000 rpm for 10 minutes to separate serum. The separated serum was inspected for haemolysis before analysis. Samples with visible haemolysis or inadequate volume were not used for biochemical estimation. Serum was analysed on the same day whenever possible. When immediate testing was not feasible, serum aliquots were stored at 2°C-8°C for short-term storage as per the manufacturer’s instructions, and repeated freeze-thaw cycles were avoided.

Serum homocysteine was estimated by enzyme-linked immunosorbent assay using a commercially available human homocysteine ELISA kit (Human Homocysteine (HCY) ELISA Kit, Cusabio Technology LLC, Houston, USA, Catalogue No.: CSB-E13814h). The assay was performed according to the manufacturer’s protocol. Before analysis, all reagents, standards, controls, and serum samples were brought to room temperature. Standards and samples were added to the microplate wells as instructed, followed by incubation, washing, enzyme conjugate reaction, substrate addition, and stop solution. The optical density was measured at 450 nm using a microplate reader (Erba LisaScan EM, Erba Mannheim, Germany). Serum homocysteine concentration was calculated from the standard calibration curve and expressed as µmol/L.

Routine biochemical investigations, including fasting blood glucose and lipid profile, were analysed using an automated clinical chemistry analyser (Beckman Coulter AU480, Beckman Coulter, Inc., Brea, California, United States). Fasting blood glucose was estimated by the hexokinase method. Total cholesterol and triglycerides were measured by enzymatic colorimetric methods. High-density lipoprotein cholesterol was measured by a direct homogeneous enzymatic method. Low-density lipoprotein cholesterol was either directly estimated or calculated using the Friedewald formula when triglyceride levels were below 400 mg/dL, according to the laboratory protocol.

Internal quality control was performed throughout the study period to maintain analytical accuracy and reproducibility. Commercially available normal and pathological control sera were analysed along with participant samples. Calibration was performed according to the manufacturer’s recommendations, and samples were processed only when quality control results were within the acceptable laboratory range. The intra-assay and inter-assay coefficients of variation for serum homocysteine estimation were maintained within acceptable limits, preferably below 10%. All biochemical analyses were performed by trained laboratory personnel following standard operating procedures.

Measurement of carotid intima-media thickness

Carotid intima-media thickness was measured using high-resolution B-mode ultrasonography with a linear-array vascular transducer. The examination was performed using an ultrasound system (LOGIQ P9, GE Healthcare, Chicago, USA) equipped with a high-frequency linear probe of 7.5-12 MHz. All scans were carried out in the radiology department under standard examination conditions.

Participants were examined in the supine position with the neck mildly extended and turned slightly away from the side being scanned. Both right and left common carotid arteries were assessed. The carotid artery was first examined in transverse and longitudinal planes to identify the common carotid artery, carotid bulb, and carotid bifurcation. Carotid intima-media thickness was measured on the far wall of the distal common carotid artery, approximately 1 cm proximal to the carotid bifurcation, at a plaque-free segment where the lumen-intima and media-adventitia interfaces were clearly visible.

For each participant, three CIMT readings were taken from the right common carotid artery and three readings from the left common carotid artery. The mean value for each side was calculated, and the average of the right and left mean CIMT values was used for final statistical analysis. Measurements were not taken over focal plaques, calcified areas, or irregular arterial segments to avoid overestimation of CIMT. In the present study, CIMT greater than 0.8 mm was considered suggestive of subclinical atherosclerosis, while CIMT of 0.8 mm or less was considered within the normal range for group classification.

All CIMT measurements were performed by an experienced radiologist trained in vascular ultrasonography. To reduce observer bias, the radiologist was blinded to the participants’ serum homocysteine levels, biochemical results, and group allocation at the time of image acquisition and interpretation. Laboratory reports were not made available to the radiologist during CIMT assessment.

To assess measurement reliability, CIMT measurements were repeated in a subset of 20 randomly selected participants. Intra-observer variability was assessed by repeating the measurements by the same radiologist after two weeks, without access to the previous readings. Inter-observer variability was assessed by a second radiologist in the same subset of participants. The intra-observer and inter-observer coefficients of variation were recorded and kept within acceptable limits. These steps were followed to improve the reliability and reproducibility of CIMT measurement.

Statistical analysis

The collected data were entered into Microsoft Excel (Microsoft Corporation, Redmond, Washington, United States) and analysed using SPSS software, version 23.0 (IBM Corp., Armonk, NY, USA). Continuous variables were expressed as mean and standard deviation, while categorical variables were presented as frequencies and percentages. The normality of continuous variables was assessed before applying parametric tests. Comparisons between the subclinical atherosclerosis group and control group were performed using the independent samples t-test for normally distributed continuous variables. Categorical variables were compared using the chi-square test or Fisher’s exact test, as appropriate.

Pearson correlation analysis was used to assess the relationship between serum homocysteine levels and CIMT. Receiver operating characteristic curve analysis was performed to evaluate the diagnostic performance of serum homocysteine in identifying subclinical atherosclerosis and to determine the optimal cut-off value. The area under the curve, sensitivity, specificity, and corresponding confidence intervals were calculated. A p-value of less than 0.05 was considered statistically significant.

Ethical considerations

The study protocol was reviewed and approved by the Institutional Ethics Committee of Malla Reddy Institute of Medical Sciences, Hyderabad, India (approval no: MRIMS/DHR-IEC-023/2024, dated: 27-12-2023), before the start of data collection. Written informed consent was obtained from all participants after explaining the study purpose, procedures, expected benefits, and possible risks in a language they could understand. Participation was voluntary, and participants were allowed to withdraw from the study at any stage without affecting their routine medical care.

Confidentiality of participant information was maintained throughout the study. Personal identifiers were removed during data entry and analysis. All procedures were carried out in accordance with accepted ethical principles for biomedical research involving human participants.

## Results

Baseline characteristics of the study population

A total of 120 participants were included, with 60 individuals in each group. The mean age in the atherosclerosis group was slightly higher compared to controls, although the difference was not statistically significant. Gender distribution was comparable between groups (Table 1).

**Table 1:** Baseline characteristics of study participants CIMT: carotid intima-media thickness.

Parameter	Group A (CIMT >0.8 mm) (n=60)	Controls (n=60)	Test statistic	p value
Age (years), mean ± SD	48.2 ± 7.1	46.5 ± 6.8	t = 1.34	0.182
Male, n (%)	38 (63.3%)	35 (58.3%)	χ² = 0.31	0.571
BMI (kg/m²), mean ± SD	26.1 ± 3.4	24.8 ± 3.1	t = 2.19	0.031
Hypertension, n (%)	22 (36.7%)	14 (23.3%)	χ² = 2.54	0.104
Diabetes mellitus, n (%)	18 (30.0%)	11 (18.3%)	χ² = 2.23	0.130

There was a modest but significant difference in BMI between the groups, with higher values observed in individuals with increased CIMT.

Comparison of serum homocysteine levels

Serum homocysteine levels were markedly elevated in individuals with early atherosclerosis compared to controls. This difference was statistically highly significant (Table 2).

**Table 2:** Comparison of serum homocysteine levels

Parameter	Group A (n=60)	Controls (n=60)	Test statistic	p value
Homocysteine (µmol/L), mean ± SEM	18.6 ± 0.67	10.9 ± 0.48	t = 9.34	<0.001

**Figure 1:**
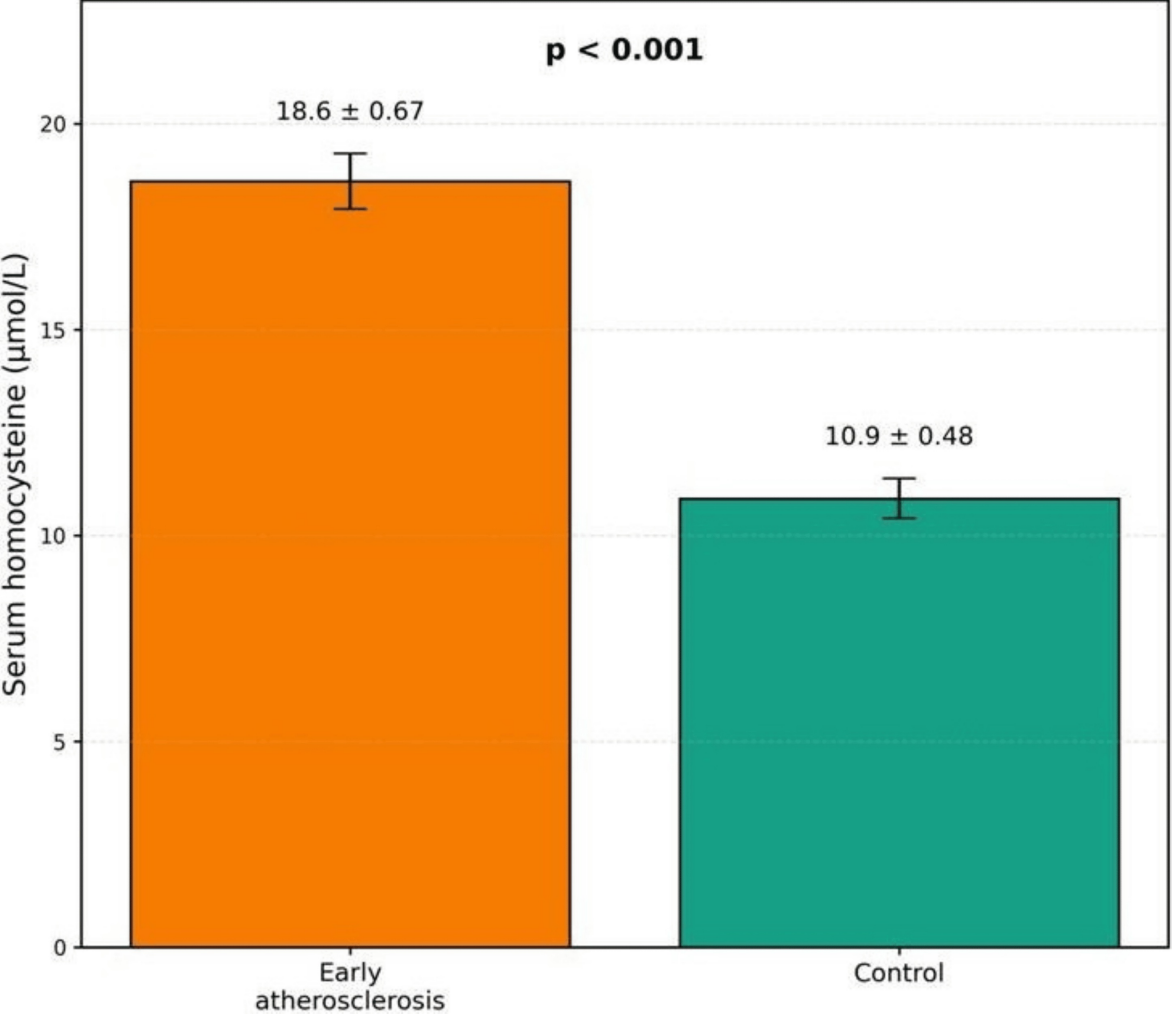
Comparison of mean serum homocysteine levels between individuals with early atherosclerosis and healthy controls

The mean homocysteine level in the study group was approximately 70% higher than in controls, indicating a strong association with early vascular changes (Figure 1).

Comparison of carotid intima-media thickness (CIMT)

CIMT values were significantly higher in the study group compared to controls, confirming the presence of early structural vascular changes (Table 3).

**Table 3:** Comparison of CIMT values CIMT: carotid intima-media thickness.

Parameter	Group A (n=60)	Controls (n=60)	Test statistic	p value
CIMT (mm), mean ± SEM	0.92 ± 0.01	0.64 ± 0.01	t = 19.80	<0.001

**Figure 2:**
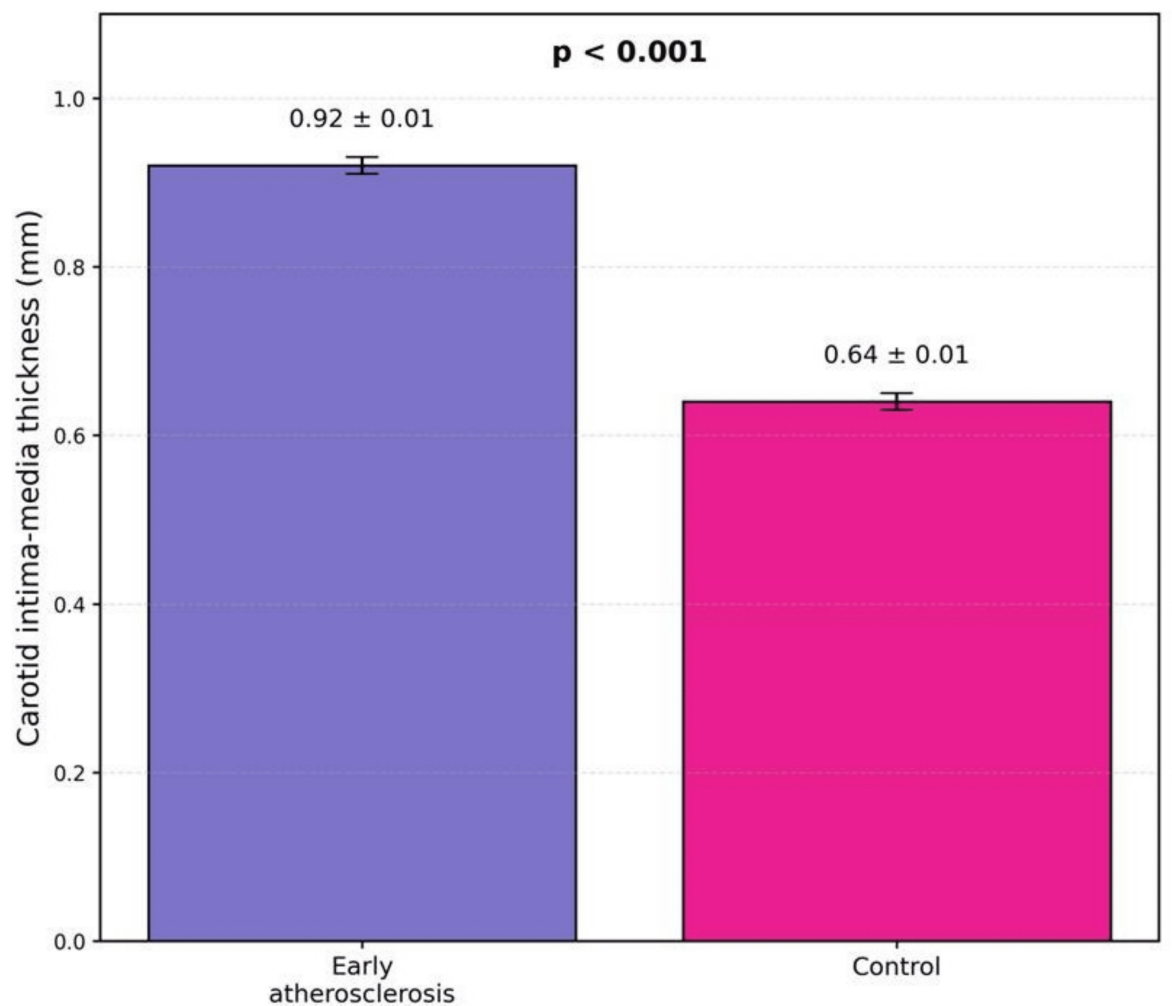
Comparison of carotid intima-media thickness (CIMT) between individuals with early atherosclerosis and healthy controls

The difference in CIMT between the groups was substantial and statistically significant, supporting the classification of cases and controls (Figure 2).

Correlation between homocysteine and CIMT

A positive correlation was observed between serum homocysteine levels and CIMT values across the study population (Table 4).

**Table 4:** Correlation analysis CIMT: carotid intima-media thickness.

Variables	Correlation coefficient (r)	p value
Homocysteine vs CIMT	0.62	<0.001

**Figure 3:**
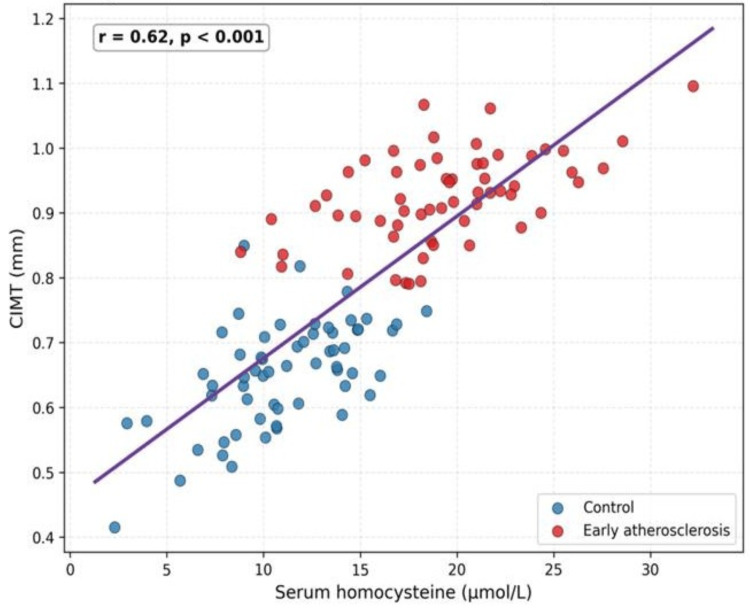
Scatter plot illustrating the relationship between serum homocysteine levels and CIMT among study participants CIMT: carotid intima-media thickness.

This indicates a moderate to strong relationship, suggesting that higher homocysteine levels are associated with increased arterial wall thickness (Figure 3).

Diagnostic performance of homocysteine

Receiver operating characteristic (ROC) analysis was performed to assess the ability of homocysteine to detect early atherosclerosis (Table 5).

**Table 5:** ROC analysis of homocysteine ROC: receiver operating characteristic.

Parameter	Value
Area under curve (AUC)	0.84
Cut-off value	14.5 µmol/L
Sensitivity	78%
Specificity	82%

**Figure 4:**
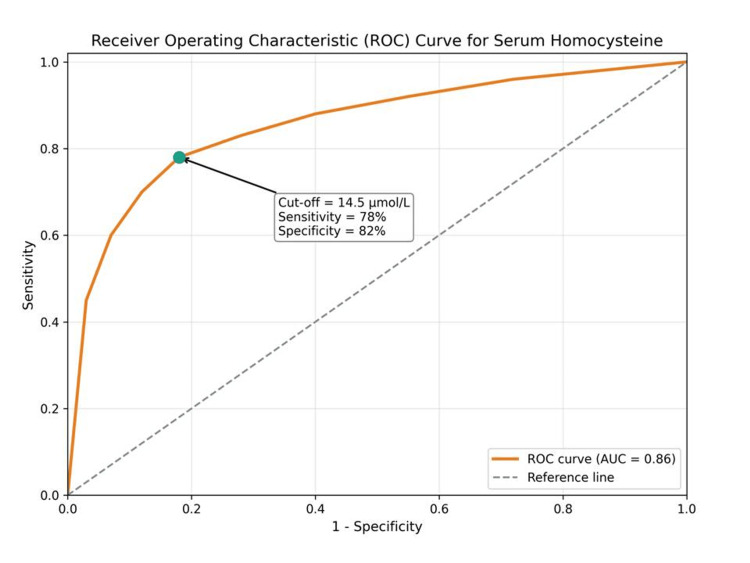
Receiver operating characteristic (ROC) curve showing the diagnostic performance of serum homocysteine in identifying early atherosclerosis AUC: area under the curve.

The AUC value indicates good diagnostic accuracy (Figure 4).

Distribution of elevated homocysteine levels

When categorized based on the cut-off value (14.5 µmol/L), a significantly higher proportion of individuals in the study group had elevated homocysteine levels.

## Discussion

The present study evaluated the role of serum homocysteine as a biomarker of early atherosclerosis by examining its relationship with carotid intima-media thickness. The findings demonstrate that individuals with early atherosclerotic changes had significantly higher serum homocysteine levels compared to healthy controls. In addition, a clear positive correlation was observed between homocysteine levels and CIMT, supporting the hypothesis that elevated homocysteine is associated with early structural changes in the arterial wall.

Atherosclerosis begins with endothelial dysfunction, which sets the stage for lipid accumulation, inflammation, and vascular remodelling. Homocysteine has been widely implicated in this process through multiple mechanisms. It promotes oxidative stress, reduces nitric oxide availability, and induces endothelial injury, thereby accelerating vascular damage [5,6]. The significantly higher homocysteine levels observed in the present study among individuals with increased CIMT align with these pathophysiological insights.

The mean serum homocysteine level in the atherosclerosis group (18.6 ± 5.2 µmol/L) was markedly elevated compared to controls (10.9 ± 3.7 µmol/L), and this difference was statistically highly significant. These findings are consistent with earlier studies that reported higher homocysteine levels in patients with subclinical and overt cardiovascular disease [8,13]. Boushey et al. demonstrated that even moderate elevations in homocysteine are associated with an increased risk of cardiovascular events [8]. Similarly, Graham et al. found a strong association between plasma homocysteine levels and vascular disease across different populations [13].

Carotid intima-media thickness is a well-established surrogate marker of early atherosclerosis and has been shown to predict future cardiovascular events [10-12]. In the present study, CIMT values were significantly higher in the study group compared to controls, confirming the presence of early vascular changes. More importantly, the observed positive correlation (r = 0.62, p < 0.001) between homocysteine and CIMT indicates that rising homocysteine levels are associated with progressive thickening of the arterial wall. Similar findings have been reported by Townend et al. (1998), who demonstrated a direct relationship between homocysteine levels and vascular structural changes [14].

The ROC analysis showed that serum homocysteine had good discriminatory ability in this study population, with an AUC of 0.84 for identifying participants with subclinical atherosclerosis. The cut-off value of 14.5 µmol/L showed a sensitivity of 78% and specificity of 82%. However, this threshold was derived from the present cohort and should not be considered a universally validated cut-off. Serum homocysteine levels may vary with dietary folate and vitamin B12 status, renal function, age, genetic background, cardiovascular risk profile, and the assay method used. Therefore, the proposed cut-off may be more applicable to this study setting than to all clinical populations. Previous reviews have suggested that homocysteine may have value as a vascular risk marker, but its independent predictive role remains uncertain. Larger multicentre studies with external validation are required before this threshold can be recommended for routine screening or risk stratification [19].

An important aspect to consider is the influence of nutritional and metabolic factors on homocysteine levels. Deficiencies of folate, vitamin B12, and vitamin B6 are known to elevate homocysteine concentrations by impairing its metabolism [15,16]. In populations where such deficiencies are common, including parts of India, homocysteine levels may be disproportionately elevated. This may partly explain the strong association observed in the present study. Therefore, interpretation of homocysteine levels should always consider the nutritional background of the population.

Although hypertension and diabetes were more frequent in the subclinical atherosclerosis group, these differences did not reach statistical significance in the present study. However, BMI showed a significant difference between the groups, indicating that conventional and metabolic risk factors may have influenced the observed association between serum homocysteine and CIMT. Therefore, the relationship between homocysteine and increased CIMT should be interpreted with caution, as it represents an unadjusted association [9]. Since multivariable analysis was not performed, the present study cannot confirm whether homocysteine is independently associated with CIMT after controlling for BMI, hypertension, diabetes, and other potential confounders. Future studies with larger sample sizes and adjusted regression models are needed to determine whether homocysteine adds predictive value beyond established cardiovascular risk factors.

From a clinical perspective, the findings of this study support the use of serum homocysteine as a simple and accessible biomarker for early detection of atherosclerosis. Its measurement is relatively inexpensive and widely available, making it suitable for routine screening in high-risk populations. Early identification of individuals with elevated homocysteine levels could allow for timely interventions, including dietary modification, vitamin supplementation, and lifestyle changes, potentially reducing the burden of cardiovascular disease.

However, it is important to note that while homocysteine is strongly associated with atherosclerosis, causality cannot be firmly established based on cross-sectional data. Some interventional trials have shown that lowering homocysteine levels with vitamin supplementation does not always translate into reduced cardiovascular events, suggesting that it may act more as a marker rather than a direct cause in certain settings [19]. Further longitudinal studies are required to clarify this relationship.

Limitations

This study has certain limitations that should be considered while interpreting the findings. First, the cross-sectional design allowed assessment of the association between serum homocysteine and carotid intima-media thickness at a single point in time, but it could not establish a causal relationship or determine the direction of this association. Second, the study was conducted at a single centre with a modest sample size, which may limit the wider applicability of the results to other populations. Third, dietary intake and serum vitamin B12, folate, and vitamin B6 levels were not assessed. Since these vitamins play an important role in homocysteine metabolism, the observed homocysteine levels in this study should be interpreted with caution, as they may have been influenced by unmeasured nutritional factors such as folate and vitamin B12 status. Fourth, multivariable analysis was not performed to adjust for potential confounders such as BMI, hypertension, diabetes, lipid parameters, and other cardiovascular risk factors. Therefore, the association between homocysteine and increased CIMT should be considered an unadjusted finding. Fifth, inflammatory and oxidative stress markers were not measured, which may have provided further insight into the biological mechanisms linking homocysteine with vascular wall changes. Finally, long-term follow-up was not performed, and therefore the study could not assess whether elevated homocysteine levels predict future cardiovascular events or progression of subclinical atherosclerosis.

## Conclusions

The present study found that serum homocysteine levels were significantly higher in adults with subclinical atherosclerosis and showed a positive association with carotid intima-media thickness. These findings suggest that homocysteine may be related to early vascular wall changes; however, this association should be interpreted cautiously because of the cross-sectional design. ROC analysis showed acceptable diagnostic performance, indicating that serum homocysteine may have value as a simple supportive marker for identifying subclinical atherosclerosis, particularly in settings where access to advanced investigations is limited. Nevertheless, the cut-off value observed in this study requires external validation, and larger prospective studies with adjustment for confounding factors are needed before homocysteine can be recommended for routine clinical risk assessment or long-term prediction of cardiovascular outcomes.
